# Associations Between Pornography Use Frequency and Intimate Partner Violence Perpetration Among Young Adult Couples: A 2-Year Longitudinal Study

**DOI:** 10.1177/08862605241234656

**Published:** 2024-03-07

**Authors:** Mandy Vasquez, Marie-Ève Daspe, Beáta Bőthe, Audrey Brassard, Yvan Lussier, Marie-Pier Vaillancourt-Morel

**Affiliations:** 1 Université du Québec à Trois-Rivières, Canada; 2 Université de Montréal, QC, Canada; 3 Université de Sherbrooke, QC, Canada

**Keywords:** pornography, intimate partner violence, young adults, couple, longitudinal

## Abstract

Pornography use is a common sexual activity for many individuals including those in a romantic relationship. Some studies have shown that violent content depicted in pornography is a risk factor for perpetration of violence in real life. Even if most of these studies examined perpetration of violent behaviors in general, not specifically toward the intimate partner, some studies have shown that pornography use frequency is related to a greater perpetration of intimate partner violence (IPV), while other studies have found that it is not significantly related. However, most previous studies were cross-sectional, sampled individuals rather than couples, and did not include separately forms of IPV (e.g., physical, psychological, and sexual). The present study examined the associations between pornography use frequency and the perpetration of physical and psychological IPV, and sexual coercion among young adult couples. A convenience sample of 113 couples aged between 16 and 29 years old completed self-reported online questionnaires two times over a 2-year period. The results of autoregressive cross-lagged models showed that a person’s pornography use frequency at Time 1 was related to their own higher sexual coercion perpetration 2 years later and that a person’s sexual coercion perpetration was related to their partner’s lower pornography use frequency 2 years later. However, no significant association between pornography use frequency and physical and psychological IPV emerged. In line with previous studies, our results suggest that pornography use represents an important risk factor for the perpetration of sexual coercion. Findings support the need to include education around pornography use in sexual violence prevention programs to avoid that young adults reproduce pornographic scripts in their sexuality.

In Western society, pornography use is a common sexual activity, which usually starts around 14 years old, and therefore is frequent in young adults including for those in a romantic relationship (Bőthe et al., 2020; Peter & Valkenburg, 2016; Vaillancourt-Morel et al., 2020). Indeed, in adult heterosexual couples, between 33.7% and 82.9% of women and between 70.5% and 91.8% of men have used pornography while in a romantic relationship (Kohut et al., 2017; Willoughby et al., 2016). Even if results on the effects of pornography use on romantic relationships are still mixed, most dyadic cross-sectional studies suggest that men’s pornography use is related to their own and their female partner’s lower relationship satisfaction and intimacy, whereas women’s use is not significantly related to their own and their male partner’s couple outcomes (Bridges & Morokoff, 2011; Poulsen et al., 2013; Willoughby & Leonhardt, 2020).

One of the components of romantic relationships that may be affected by pornography use is the perpetration of violence toward the romantic partner, namely intimate partner violence (IPV). Indeed, as even mainstream pornography depicts violence against women (Carrotte et al., 2020), pornography users may gradually become desensitized to aggression and reproduce it with their romantic partner. Yet, scientific evidence on the associations between pornography use and IPV perpetration yielded contradictory findings with some studies reporting significant positive associations (Beymer et al., 2021; Brem et al., 2021), whereas other studies supporting that pornography use is not significantly related to any form of IPV perpetration (Hatch et al., 2020; Herbitter et al., 2022). However, these previous studies have been largely cross-sectional, relied mostly on samples of individuals with low generalizability to the general population (e.g., men arrested for IPV, American army soldiers, and sexual minority individuals) rather than couples from the community, and were limited to the examination of one form of IPV. The current study examined the associations between pornography use frequency and three forms of IPV perpetration among young adult couples from the community using a dyadic and longitudinal design. Indeed, as young adults are frequent pornography users (Bőthe et al., 2020; Peter & Valkenburg, 2016) and are at a turning point in their development of relational skills, they may particularly benefit from porn literacy and dating violence prevention.

## IPV Perpetration

IPV perpetration refers to physical, sexual, and psychological harm committed toward a romantic partner (Breiding et al., 2015). The most common form of IPV is psychological violence, which refers to using communication with the intent to harm mentally or emotionally, or exert control over a partner (e.g., threatening a partner; Breiding et al., 2015; Straus et al., 1996). Almost half of women (47.1%) and men (47.3%) reported experiencing psychological IPV in their lifetime (Smith et al., 2017). Physical IPV refers to using physical force with the potential of causing harm to a partner (e.g., slap a partner), while sexual IPV refers to any sexual behavior made without consent or threat or coercion aimed at inciting the partner to engage in sexual activity (e.g., using threats to make a partner have sex; Breiding et al., 2015; Straus et al., 1996). Thus, sexual IPV includes sexual coercion, which refers to any behaviors to compel the partner to engage in unwanted sexual activity (e.g., insisting on having sex when the partner does not want to; Straus et al., 1996). Approximately one-third of women (37.3%) and men (30.9%) have experienced physical and/or sexual IPV (Smith et al., 2017). Perpetration of IPV is particularly prevalent in young adults (i.e., aged 16–29 years old) as 55% of them report having perpetrated at least one form of IPV in the past year (Cotter, 2021; Emond et al., 2023). Indeed, young adults may be more at risk to experience frequent conflicts in their romantic relationships that escalate into perpetration of IPV as they are often experiencing their first serious romantic relationship which is often marked by couple disagreements. A better understanding of risk factors of IPV perpetration in young adult couples is an important step in prevention efforts.

### Pornography Use and IPV Perpetration

As most pornographic materials, even mainstream ones, depict violence (Bridges et al., 2010; Carrotte et al., 2020), pornography use has been identified as an important risk factor of IPV among adolescents and young adults (Rostad et al., 2019). The association between pornography use and IPV perpetration is in line with the Sexual Script Theory (Brem et al., 2021; Rostad et al., 2019), which suggests that sexual scripts are learned from the observation of others and exposure to mass media including pornography, and then teach one how to recognize sexual situations, and how to behave in sexual encounters (Bridges et al., 2016; Simon & Gagnon, 1986). Mainstream pornography often represents men in dominant position, degrading or humiliating women, and consent is rarely at the forefront in the scenario depicted (Sun et al., 2016). Considering the sexual nature of these scripts, some users might be tempted to reproduce them in their sexual life (Bridges et al., 2016), and it may be particularly true for young adults that often use pornography as a source of sexual education (Sun et al., 2016). Thus, young adult users, particularly men, may develop coercive sexual scripts and therefore be more at the risk of perpetrating sexual coercion (Bridges et al., 2016; Marshall & Miller, 2023). The scripts portrayed in pornography may also be related to how a person acts with their partner outside of sexuality. A study found that in over 304 pornographic scenes analyzed, 88.2% depicted physical aggressions such as spanking and slapping, while 48.7% contained verbal aggressions (e.g., name-calling; Bridges et al., 2010). Therefore, being exposed to these behaviors in pornography may be a risk factor of physical and psychological IPV perpetration particularly for men. Indeed, the more a person is exposed to pornography, the more this person may integrate these gendered behaviors in their view of others, and the higher would be the risk of reproducing these behaviors in their real-life interactions (Sun et al., 2016).

A handful of cross-sectional studies examined the associations between pornography use and IPV perpetration. In a cross-sectional study among 7,346 American army soldiers (10.1% women), soldiers who used pornography had a higher risk of reporting IPV perpetration compared to those who had never used pornography in their life (Beymer et al., 2021). In a cross-sectional study among 273 men arrested for IPV and enrolled in a battered intervention program, problematic pornography use (i.e., excessive, compulsive, or uncontrollable use) was associated with more perpetration of sexual IPV and physical IPV (Brem et al., 2021). In a cross-sectional study among 2,830 American adolescents, violent pornography use was associated with a 1.77 greater odd of perpetrating threatening IPV in women, whereas it was associated with a 3.34 greater odd of perpetrating sexual IPV in men (Rostad et al., 2019). However, in a sample of 176 American adolescent sexual minority girls, violent pornography use was not significantly associated with IPV perpetration (adjusted odds ratio = 1.28) (Herbitter et al., 2022). Overall, pornography use seems to be associated with all three forms of IPV perpetration, with men more likely to perpetrate sexual IPV, and women more likely to perpetrate psychological IPV. However, these studies used samples that may not represent the experience of the general population (e.g., army soldiers, violent men, and girls from a sexual minority) and most examined exclusively problematic or violent pornography use instead of pornography use frequency in general. Moreover, these studies used a cross-sectional design. Thus, we know little about the direction of the associations, i.e., whether pornography use predicts perpetration of IPV, perpetration of IPV predicts pornography use, or whether these associations are bidirectional.

Few longitudinal studies examined the associations between pornography use and IPV perpetration. In a longitudinal study among 892 American university students (80.5% women), pornography use frequency did not predict the perpetration of physical IPV 3 months later and vice versa for both men and women (Hatch et al., 2020). In another longitudinal study among 1,234 Americans, an increase in solitary pornography use was associated with greater perpetration of psychological IPV over a 20 months period of time, while it was not significantly related to physical IPV perpetration (Huntington et al., 2021). Unfortunately, these studies had either short duration or did not include all three forms of IPV perpetration (Hatch et al., 2020; Huntington et al., 2021). Moreover, they sampled individuals rather than couples, precluding the examination of dyadic associations. Yet, some studies have found that a person’s pornography use is related to their partner’s outcomes (e.g., sexual dissatisfaction, communication difficulties, and sexual dysfunction; Bőthe et al., 2021; Szymanski & Stewart-Richardson, 2014).

The only study among a sample of couples (Jongsma & Timmons Fritz, 2021), allowing to examine partner effects (i.e., how a person’s pornography use is related to their partner perpetration of IPV) showed that, among a sample of 132 Canadian different-sex couples, higher pornography use frequency at baseline in men predicted increases in their own and their partner perpetration of IPV over 4 months. However, no significant associations were found in women (Jongsma & Timmons Fritz, 2021). This study followed couples only over 4 months, whereas a longer follow-up may increase the odds of IPV perpetration as it often appears over time in romantic relationships. Moreover, this study combines the three forms of IPV, precluding to understand if pornography use is related to each form of IPV perpetration or solely to sexual IPV perpetration given the sexual nature of pornography.

### Current Study

The overall aim of the current study was to examine the associations between pornography use frequency and three different forms of IPV perpetration among young adult couples using a dyadic and longitudinal design. The current study goes beyond previous investigations regarding the links between pornography use and IPV by (a) using a sample of young adults from the community, (b) examining the directionality of associations using a 2-year longitudinal design, and (c) using a dyadic design considering both partners’ pornography use and IPV perpetration. Specifically, the first aim was to examine the directionality of the associations between a person’s pornography use frequency and their own and their partner’s physical and psychological IPV, and sexual coercion perpetration over a 2-year period. We hypothesized that a person’s pornography use frequency would be related to their own higher physical and psychological IPV, and sexual coercion perpetration 2 years later. The partner effects were examined in an exploratory way. The second aim was to examine whether the association between a person’s pornography use frequency and their own and their partner’s physical and psychological IPV, and sexual coercion perpetration were different between women and men. As pornography use and sexual coercion are more prevalent and frequent in men (Bridges et al., 2016; Smith et al., 2017), sexual scripts depicted in pornography are highly gendered (i.e., dominant men and submissive women; Bridges et al., 2016; Sun et al., 2016), and the only study comparing the pornography–IPV association between men and women reported significant associations for men only (Jongsma & Timmons Fritz, 2021), we hypothesized that only men’s pornography use frequency would be related to their own higher physical and psychological IPV, and sexual coercion perpetration 2 years later. Significant differences between women and men in the partner effects were also examined in an exploratory way. Given that pornography use and masturbation are highly correlated, we controlled for masturbation frequency. Based on previous study (Grubbs et al., 2019), we controlled for religiosity as it may be related to how a person perceives their pornography use. We also controlled for relationship length as IPV may evolve the course of a romantic relationship in young adult couples (Manning et al., 2018).

## Method

### Participants

A total of 113 young adult couples (*M_age_* = 23.38 years, *SD* = 2.69, ranged between 18 and 29) were included in this study. Out of these couples, 106 were mixed-sex couples (94.0%) and 7 were same-sex couples (6.0%; one man-man and six woman-woman couples). In terms of relationship status, 33.6% (*n* = 38) of the couples did not live together, 64.6% (*n* = 73) cohabited, and 1.8% (*n* = 2) were married. Couples were together for an average of 2.95 years (*SD* = 2.27; ranged between 0.08 and 9.33 years). Regarding their cultural identity, most participants (88.5%; *n* = 200) reported being French Canadian, 4.9% (*n* = 11), Western European, 0.9% (*n* = 2), Eastern European, 1.8% (*n* = 4), English Canadian, and 3.9% (*n* = 9) reported other cultural identities (i.e., Asian, Latin American/South American, and Caribbean).

### Procedure

This study was part of a larger longitudinal research advertised as a study about digital technologies and intimate relationships. Recruitment was carried out using online advertisements on various web pages, social media (e.g., Facebook, Instagram), and email lists. To be eligible, participants had to be aged between 16 and 29 years old and understand French. Eligible participants were then redirected to the consent form and anonymous survey (Time 1) on a secure online survey software (Qualtrics Research Suite, 2019). For the current study, only participants that were currently in a romantic relationship and that both members of the couple completed the survey at Time 1 were included. Three attention questions were distributed within the survey. Participants who failed at least two of these questions were excluded. Two years later (Time 2), partners completed the survey again. As compensation, each partner received CAN$10 after completing Time 1, and CAN$15 after completing Time 2. All procedures were approved by the Université du Québec à Trois-Rivières’ Institutional Review Board.

Recruitment took place between January and October 2019. Of the 1,508 interested participants that started the eligibility survey, 1,384 (91.8%) were eligible for the larger project, gave their informed consent, and were directed to the online survey. Of these, 19 (1.4%) failed at least two out of three attention testing questions and 364 (26.3%) dropped out during Time 1, resulting in 1,001 (72.3%) participants. Out of these participants, 306 (30.6%, 153 couples) were eligible for the present study; they were currently in a relationship and both partners completed Time 1. Of the 153 couples that completed Time 1, 40 (26.1%) were excluded as a result of separating before Time 2, resulting in a final sample of 113 couples (226 participants). Of these 226 participants, 201 participated in Time 2, for a completion rate of 88.9% (90 of the 113 couples where both partners completed Time 2 for a completion rate of 79.6%). Missing data due to attrition were estimated using full information maximum likelihood (FIML).

### Measures

#### Pornography Use Frequency

We provided the following definition of pornography to participants (Kohut et al., 2017): “For the following questions, the term ‘pornography’ is used to refer to: intentionally looking at or listening to on an electronic device (e.g., cellphone, computer, Ipad): (a) pictures or videos of nude individuals, or (b) pictures or videos in which people are having sexual activities.” Then, pornography use frequency in the past 3 months was assessed with one question: “On average, in the last THREE months, how many times did you use pornography?” Participants responded on an eight-point scale ranging from 0 = *never*, to 7 = *many times per day*.

#### Intimate Partner Violence

IPV perpetration in the last year was assessed using nine items from the Revised Conflict Tactics Scale (CTS2; Straus et al., 1996). In line with past short forms of the CTS2 (Hébert & Parent, 2000; Straus & Douglas, 2004), the items with the highest correlations from the CTS2 were selected and augmented by adding behaviors measured by the unselected items in the scale. Each form of violence (psychological, physical, and sexual coercion) was measured using three items. Participants were asked to report the frequency with which they had perpetrated psychological (i.e., “I insulted, swore at, shouted at, or yelled at my partner,” “I destroyed something belonging to my partner,” and “I threatened to hit or throw something at my partner”) and physical (i.e., “I threw something at my partner that could hurt,” “I pushed, shoved, or slapped my partner,” and “I punched, kicked, or hit my partner with something that could hurt”) violent behaviors, and sexual coercion (i.e., “I insisted to have sex when my partner did not want to [but did not use physical force],” “I used threats to make my partner have sex,” and “I used force [like hitting, holding down, or using a weapon] to make my partner have sex”) toward their romantic partner in the past year on a seven-point scale ranging from 0 = *Never* to 6 = *More than 20 times*. Total scores were computed by averaging the three items for each subscale. The ordinal alpha coefficient using polychoric correlations was adequate for both psychological (α_T1_ = .89; α_T2_ = .88) and physical violence (α_T1_ = .98; α_T2_ = .97). Due to the low prevalence of sexual coercion in our sample and as there was no variability in two items at Time 2 (i.e., no participants reported that they used threats to make their partner have sex or used force [like hitting, holding down, or using a weapon] to make their partner have sex in the last year), sexual coercion was recoded into a dichotomous variable (i.e., 0 = *no sexual coercion perpetration* and 1 = *sexual coercion perpetration* if any of the item was endorsed).

#### Control Variables

Participants’ masturbation frequency in the past 3 months was assessed with one question: “In the last THREE MONTHS, how many times did you masturbate?” Participants responded on a nine-point scale ranging from 0 = *not at all*, to 8 = *more than 1 time per day*.

Religiosity was measured using the Religious Engagement Subscale of the Religiosity Inventory (RES; Pennycook et al., 2012). The RES assesses religious engagement or level of participation and consists of three questions (e.g., *How important was religion in your daily life?*). Participants reported their answers on various six-point scales ranging from 1 = *irrelevant or never* to 6 = *highest importance or more than once a week*. A higher total score indicated a higher level of religiosity. Internal consistency was adequate in the present sample (α_T1_ = .83).

Relationship length was assessed with one question: “How long, in months, have you been in your current romantic relationship?” Even though partners’ responses were strongly correlated (*r* = .99, *p* < .001), some minor differences naturally occurred. Therefore, a mean score for relationship length was calculated from both partners’ answers.

### Statistical Analyses

Descriptive statistics, paired-samples *t*-tests, and correlations were computed in SPSS 28 (IBM Corp., 2020). Mplus 8.7 (Muthén & Muthén, 2020) was used to test the associations between each partner’s pornography use frequency and IPV perpetration (i.e., physical and psychological IPV, and sexual coercion), controlling for masturbation frequency, religiosity, and relationship length. To account for the nonindependence between partners and to examine the direction of the associations between the examined variables, three autoregressive cross-lagged models were conducted within an actor-partner interdependence framework (APIM; Kenny et al., 2006). The robust maximum likelihood estimator was used to take into account the naturally non-normal distribution of the data. The FIML method was used to account for missing data (ranging from 0% for variables at Time 1 to 11.9% for IPV at Time 2). Models were evaluated taking into consideration commonly used goodness-of-fit indices (Marsh et al., 2005; Schermelleh-Engel et al., 2003): the Comparative Fit Index (CFI; ≥0.90 adequate; ≥0.95 good), the Tucker–Lewis index (TLI; ≥0.90 adequate; ≥0.95 good), and the root-mean-square error of approximation (RMSEA; ≤0.08 adequate, ≤0.05 good).

This study combined mixed-sex (*n* = 106 couples) and same-sex couples (*n* = 7 couples). For mixed-sex couples, theoretically, partners were expected to be distinguishable by their sex, which was verified by the omnibus within-dyad tests of distinguishability (Kenny et al., 2006). These chi-square tests constrained means, variances, and covariances to be equal across men and women, with a significant *p*-value indicating that the pattern of means, variances, and covariances differed significantly between women and men. As the omnibus within-dyad tests of distinguishability were significant, men and women in mixed-sex couples were considered distinguishable in all models: physical IPV model: χ^2^(48) = 406.34, *p* < .001; psychological IPV model: χ^2^(48) = 294.246, *p* < .001; sexual coercion model: χ^2^(48) = 211.222, *p* < .001. Moreover, to determine if the examined associations in the models were significantly different between men and women, an unconstrained model was compared to a model in which all paths were constrained to be equal between men and women using corrected chi-square difference tests, with a significant *p*-value indicating that the associations differ significantly between men and women. Considering that mixed-sex couples were considered distinguishable, which is impossible for same-sex couples (i.e., sex could not distinguish partners within a dyad), and that only seven same-sex couples were included in the sample, it was not possible to conduct the same analysis in same-sex couples (i.e., due to the low levels of variance in the responses and the low sample size). Nonetheless, taking into account ethical considerations, we decided not to exclude these participants from the sample and provided preliminary results.

## Results

### Preliminary Results in Same-Sex Couples

Descriptive statistics and correlations between pornography use frequency, physical IPV perpetration, psychological IPV perpetration, sexual coercion perpetration, and control variables in same-sex couples are shown in Supplemental Table S1. Most of the correlations were not significant as a result of the underpowered nature of the data (*n* = 7 couples). Therefore, we discuss these preliminary results based on their effect sizes (i.e., ≥ |.10| is small, ≥ |.30| is moderate, and ≥|.50| is strong), and results should be interpreted with caution (Cohen, 1992). A person’s pornography use frequency at Time 1 had strong, positive significant associations with their own masturbation frequency at Time 1 and their pornography use frequency at Time 2. A person’s psychological IPV perpetration at Time 1 had a strong, negative significant association with their own pornography use frequency at Time 2. A person’s masturbation frequency at Time 1 had a strong, positive significant association with their partner’s physical IPV perpetration at Time 1. A person’s religiosity at Time 1 had a strong, positive significant association with their partner’s religiosity at Time 1.

### Descriptive and Correlational Results in Mixed-Sex Couples

Descriptive statistics and comparisons of women and men’s scores in mixed-sex couples are presented in Table 1. Significant differences were observed between women and men regarding their pornography use frequency and masturbation frequency at both Time 1 and Time 2 with strong effect sizes, with men reporting higher scores than women. Regarding physical and psychological IPV perpetration, there were no significant differences between women and men at Time 1 and Time 2. Men reported significantly greater sexual coercion perpetration than women at Time 2 with a small effect size, but this difference was not significant at Time 1.

**Table 1.** Descriptive Statistics for Pornography Use Frequency, IPV Perpetration, and Control Variables, in Men and Women in Mixed-Sex Couples (*n* = 106).

	Men		Women		*t* (*df*)	*p*	*d*
Variables	*M* (*SD*)	Observed Range	*M (SD)*	Observed Range			
Pornography use frequency T1^a^	3.83 (1.62)	0–7	1.38 (1.48)	0–5	−12.78 (105)	<.001	1.97
Physical IPV perpetration T1	0.11 (0.50)	0–4	0.40 (2.23)	0–18	1.58 (105)	.116	1.84
Psychological IPV perpetration T1	0.88 (1.36)	0–6	1.20 (2.65)	0–18	1.55 (105)	.124	2.13
Sexual coercion perpetration T1	0.17 (0.38)	0–1	0.10 (0.31)	0–1	−1.41 (105)	.163	0.48
Masturbation frequency T1	4.67 (1.73)	0–8	2.71 (2.11)	0–8	−8.02 (105)	<.001	2.52
Religiosity T1	4.50 (2.67)	3–17	4.47 (2.36)	3–17	−0.13 (105)	.895	2.20
Pornography use frequency T2^a^	3.73 (1.83)	0–6	1.38 (1.57)	0–5	−9.85 (84)	<.001	2.20
Physical IPV perpetration T2	0.05 (0.26)	0–2	0.15 (1.20)	0–11	0.87 (84)	.387	1.12
Psychological IPV perpetration T2	0.72 (1.26)	0–5	1.05 (1.92)	0–13	1.49 (84)	.140	2.04
Sexual coercion perpetration T2	0.14 (0.35)	0–1	0.04 (0.19)	0–1	−2.58 (84)	.012	0.38

*Note: df* = degree of freedom; IPV = intimate partner violence; *M* = Mean; *SD* = Standard Deviation; T1 = Time 1; T2 = Time 2.

a 0 = never, 1 = less than 1 time per month, 2 = 1 time per month, 3 = 2–3 times per month, 4 = 1 time per week, 5 = many times per week, 6 = 1 time per day, and 7 = many times per day.

Correlations between all study variables are shown in Table 2. For the significant associations regarding the main study variables, men’s pornography use frequency at Time 1 had moderate, negative significant association with their own physical IPV perpetration at Time 1. Men’s physical IPV perpetration at Time 1 had moderate, positive significant associations with their own psychological IPV and sexual coercion perpetration at Time 1, and their own physical IPV perpetration at Time 2, as well as strong, positive significant associations with their partner’s physical and psychological IPV perpetration at Time 1 and Time 2. Women’s physical IPV perpetration at Time 1 had strong, positive significant associations with their own psychological IPV perpetration at Time 1 and Time 2, and their own physical IPV perpetration at Time 2, as well as moderate, positive significant associations with their partner’s psychological IPV and sexual coercion perpetration at Time 1, and their partner’s physical IPV perpetration at Time 2. Men’s psychological IPV perpetration at Time 1 had moderate, positive significant associations with their own sexual coercion perpetration at Time 1, and their own physical and psychological IPV perpetration at Time 2, as well as strong, positive significant associations with their partner’s psychological IPV perpetration at Time 1 and Time 2, and a small, positive significant association with their partner’s sexual coercion perpetration at Time 1, and their partner’s physical IPV perpetration at Time 2. Women’s psychological IPV perpetration at Time 1 had strong, positive significant associations with their own physical and psychological IPV perpetration at Time 2, a small, positive significant association with their own sexual coercion perpetration at Time 1, as well as moderate, positive significant associations with their partner’s sexual coercion perpetration at Time 1 and physical IPV perpetration at Time 2. Men’s sexual coercion perpetration at Time 1 had small, positive significant associations with their partner’s physical and psychological IPV perpetration at Time 2. Women’s sexual coercion perpetration at Time 1 had a strong, positive significant association with their own sexual coercion perpetration at Time 2. Men’s physical IPV perpetration at Time 2 had moderate, positive significant associations with their own psychological IPV perpetration at Time 2, and their partner’s physical IPV and psychological IPV perpetration at Time 2. Women’s physical IPV perpetration at Time 2 had a strong, positive significant association with their own psychological IPV perpetration at Time 2. Finally, men’s psychological IPV perpetration at Time 2 had a small, positive significant association with their partner’s psychological IPV perpetration at Time 2.

**Table 2.** Correlations Between Pornography Use Frequency, IPV Perpetration, and Control Variables in Mixed-Sex Couples (*n* = 106).

Variables	Skew. (*SE*)	Kurt. (*SE*)	1.	2.	3.	4.	5.	6.	7.	8.	9.	10.	11.
1. M’s Pornography use frequency T1	−0.83 (0.24)	0.12 (0.47)	—										
2. W’s Pornography use frequency T1	0.75 (0.24)	−0.71 (0.47)	.19	—									
3. M’s Physical IPV perpetration T1	5.68 (0.24)	37.02 (0.47)	−.32**	.01	—								
4. W’s Physical IPV perpetration T1	7.01 (0.24)	50.22 (0.47)	−.18	.05	.82**	—							
5. M’s Psychological IPV perpetration T1	1.67 (0.24)	2.28 (0.47)	−.11	−.07	.47**	.46**	—						
6. W’s Psychological IPV perpetration T1	5.05 (0.24)	29.78 (0.47)	−.12	.01	.77**	.90**	.60**	—					
7. M’s Sexual coercion perpetration T1	1.78 (0.24)	1.21 (0.47)	−.05	−.05	.35**	.38**	.30**	.39**	—				
8. W’s Sexual coercion perpetration T1	2.64 (0.24)	5.04 (0.47)	.06	−.07	.05	.15	.19*	.24*	.01	—			
9. M’s Masturbation frequency T1	−0.89 (0.24)	0.65 (0.47)	.79**	.17	−.22*	−.11	−.10	−.07	−.07	.08	—		
10. W’s Masturbation frequency T1	0.37 (0.24)	−0.76 (0.47)	.01	.51**	.27**	.35**	.11	.28**	.05	.05	.15	—	
11. M’s Religiosity T1	2.76 (0.24)	8.31 (0.47)	−.03	−.08	−.09	−.04	−.01	.01	.28**	−.12	−.10	−.06	—
12. W’s Religiosity T1	2.49 (0.24)	8.59 (0.47)	−.09	−.04	.07	.13	.10	.16	.18	.04	−.08	−.05	.62**
13. Relationship length in months T1	0.86 (0.24)	−0.12 (0.47)	.09	.01	<−.01	.05	.14	.16	.15	−.05	.15	−.11	.13
14. M’s Pornography use frequency T2	−1.03 (0.25)	−0.04 (0.50)	.67**	.19	−.12	−.14	.04	−.04	−.08	−.16	.57**	.04	−.09
15. W’s Pornography use frequency T2	0.74 (0.25)	−0.83 (0.49)	.17	.65**	−.01	.01	−.10	−.01	−.20	.06	.19	.50**	−.06
16. M’s Physical IPV perpetration T2	6.37 (0.25)	43.16 (0.50)	−.12	.16	.48**	.39**	.33**	.45**	.18	−.05	−.14	.16	.12
17. W’s Physical IPV perpetration T2	9.51 (0.25)	92.30 (0.49)	−.13	.10	.80**	.97**	.26*	.82**	.22*	−.04	−.11	.25*	−.05
18. M’s Psychological IPV perpetration T2	1.80 (0.25)	2.41 (0.50)	.03	−.01	.08	−.05	.48**	.09	.06	−.03	−.10	−.01	.19
19. W’s Psychological IPV perpetration T2	2.90 (0.25)	11.67 (0.49)	−.12	.06	.58**	.64**	.54**	.79**	.29**	−.01	−.05	.18	.13
20. M’s Sexual coercion perpetration T2	1.95 (0.25)	1.85 (0.50)	.16	−.17	−.09	−.03	.16	.03	.17	.19	.15	−.10	.11
21. W’ Sexual coercion perpetration T2	5.50 (0.25)	28.90 (0.49)	.10	.15	−.04	−.03	.02	.13	−.08	.56**	.01	−.01	−.05
	12.	13.	14.	15.	16.	17.	18.	19.	20.				
13. Relationship length in months T1	.11	—											
14. M’s Pornography use frequency T2	−.07	−.04	—										
15. W’s Pornography use frequency T2	−.05	−.10	.17	—									
16. M’s Physical IPV perpetration T2	.14	.21*	−.20	−.04	—								
17. W’s Physical IPV perpetration T2	.03	.12	−.12	.04	.39**	—							
18. M’s Psychological IPV perpetration T2	.03	.01	.05	−.08	.38**	−.07	—						
19. W’s Psychological IPV perpetration T2	.26*	.26**	−.05	.09	.37**	.64**	.23*	—					
20. M’s Sexual coercion perpetration T2	.01	−.10	.17	−.16	−.07	−.05	.11	.04	—				
21. W’ Sexual coercion perpetration T2	−.08	.08	.10	.10	−.03	−.02	−.06	.01	.11				

*Note:* IPV = intimate partner violence; Kurt. = Kurtosis; M = Men; Skew. = Skewness; T1 = Time 1; T2 = Time 2; W = Women.

* *p* < .05. **p < .01.

### Pornography Use Frequency and IPV Perpetration in Mixed-Sex Couples

Results of all three models autoregressive cross-lagged APIMs are presented in Table 3.

**Table 3.** Autoregressive Cross-Lagged Models of Pornography Use Frequency and IPV Perpetration in Mixed-Sex Couples (*n* = 106).

Variables	*B(SE)*	*p*	β	95% CI		*B(SE)*	*p*	β	95% CI	
				Lower	Upper				Lower	Upper
	Physical IPV perpetration T2					Pornography use frequency T2				
Actor’s pornography use frequency T1	0.03 (0.02)	.081	.09	<−0.01	0.06	**0.59 (0.09)**	**< .001**	**.54**	**0.42**	**0.77**
Partner’s pornography use frequency T1	0.02 (0.01)	.128	.07	−0.01	0.05	0.09 (0.09)	.310	.08	−0.08	0.26
Actor’s physical IPV perpetration T1	**0.59 (0.04)**	**<.001**	**1.00**	**0.52**	**0.67**	−0.06 (0.04)	.133	−.05	−0.13	0.02
Partner’s physical IPV perpetration T1	**−0.07 (0.02)**	**<.001**	**−.26**	**−0.10**	**−0.04**	−0.04 (0.05)	.338	−.03	−0.14	0.05
Actor’s masturbation frequency T1	−0.01 (0.01)	.373	−.05	−0.04	0.02	**0.17 (0.07)**	**.012**	**.20**	**0.04**	**0.31**
Partner’s masturbation frequency T1	−0.01 (0.01)	.422	−.04	−0.03	0.01	0.01 (0.07)	.107	.01	−0.13	0.14
Actor’s religiosity T1	**0.02 (0.01)**	**.032**	**.09**	**<0.01**	**0.03**	0.01 (0.04)	.752	.02	−0.07	0.10
Partner’s religiosity T1	−0.02 (0.01)	.142	−.08	−0.03	0.01	0.03 (0.05)	.484	.05	−0.06	0.12
Relationship length in months	<−0.01 (<0.01)	.052	.08	**<0.01**	< 0.01	<−0.01 (<0.01)	.301	−.05	−0.01	−0.14
	Psychological IPV perpetration T2					Pornography use frequency T2				
Actor’s pornography use frequency T1	0.06 (0.07)	.377	.05	−0.07	0.18	**0.60 (0.09)**	**<.001**	**.55**	**0.42**	**0.78**
Partner’s pornography use frequency T1	−0.03 (0.06)	.571	−.04	−0.15	0.09	0.09 (0.09)	.299	.09	−0.08	0.27
Actor’s psychological IPV perpetration T1	**0.69 (0.05)**	**<.001**	**.73**	**0.60**	**0.78**	0.01 (0.06)	.923	<.01	−0.10	0.11
Partner’s psychological IPV perpetration T1	−0.04 (0.09)	.693	−.04	−0.21	0.14	−0.01 (0.06)	.838	−.02	−0.13	0.10
Actor’s masturbation frequency T1	0.03 (0.05)	.604	.03	−0.07	0.13	**0.15 (0.07)**	**.028**	**.17**	**0.02**	**0.28**
Partner’s masturbation frequency T1	0.02 (0.05)	.723	.02	−0.08	0.12	−0.02 (0.07)	.826	−.02	−0.15	0.12
Actor’s religiosity T1	**0.20 (0.05)**	**<.001**	**.29**	**0.10**	**0.29**	−0.01 (0.04)	.849	−.01	−0.09	0.08
Partner’s religiosity T1	−0.08 (0.05)	.132	−.12	−0.19	0.02	0.01 (0.04)	.761	.02	−0.07	0.10
Relationship length in months	<.01 (<.01)	.982	<.01	−0.01	0.01	−0.01 (<0.01)	.094	−.10	−0.01	< 0.01
	Sexual coercion perpetration T2					Pornography use frequency T2				
Actor’s pornography use frequency T1	**0.03 (0.01)**	**.012**	**.20**	**0.01**	**0.05**	**0.57 (0.09)**	**< .001**	**.52**	**0.39**	**0.75**
Partner’s pornography use frequency T1	<0.01 (0.01)	.716	.03	−0.02	0.03	0.09 (0.08)	.292	.09	−0.08	0.25
Actor’s sexual coercion perpetration T1	**0.35 (0.12)**	**.003**	**.48**	**0.12**	**0.58**	0.36 (0.37)	.320	.08	−0.35	1.08
Partner’s sexual coercion perpetration T1	0.01 (0.03)	.726	.02	−0.05	0.08	**−0.94 (0.36)**	**.009**	**−.20**	**−1.64**	**−0.24**
Actor’s masturbation frequency T1	−0.01 (0.01)	.322	−.05	−0.02	0.01	**0.16 (0.07)**	**.016**	**.19**	**0.03**	**0.30**
Partner’s masturbation frequency T1	−0.01 (0.01)	.150	−.10	−0.03	0.01	< −0.01 (0.06)	.939	−.01	−0.12	0.11
Actor’s religiosity T1	−0.01 (0.01)	.158	−.12	−0.03	< 0.01	−0.04 (0.04)	.361	−.06	−0.12	0.05
Partner’s religiosity T1	0.01 (0.01)	.204	.08	<−0.01	0.02	0.07 (0.05)	.172	.10	−0.03	0.16
Relationship length in months	<0.01 (<0.01)	.349	.03	<0.01	<0.01	−0.01 (<0.01)	.196	−.08	−0.01	< 0.01

*Note*: Significant associations at *p* < .05 are presented in bold. IPV = intimate partner violence; T1 = Time 1; T2 = Time 2.

#### Physical IPV Perpetration

The corrected chi-square difference test (χ^2^[18] = 28.61, p = .053) indicated no significant difference between the unconstrained and the constrained models, suggesting that the associations do not differ significantly between men and women. Therefore, for the sake of parsimony, we reported the results of the constrained model. The fit indices of this constrained model were acceptable for the physical IPV perpetration model, χ^2^[18] = 28.61, p = .053; CFI = 0.989; TLI = 0.975; RMSEA = 0.075, 90% CI [< 0.001, 0.124]. A person’s prior pornography use frequency (Time 1) was positively and strongly associated with their own later pornography use frequency (Time 2). A person’s prior physical IPV perpetration (Time 1) was positively and strongly related to their own later physical IPV perpetration (Time 2). Regarding partner effects, a person prior physical IPV perpetration (Time 1) was negatively associated with their partner’s physical IPV perpetration 2 years later (Time 2) with a strong effect size. However, a person’s prior pornography use frequency (Time 1) was not significantly related to their own and their partner’s physical IPV perpetration 2 years later (Time 2). A person’s prior physical IPV perpetration (Time 1) was not significantly related to their own and their partner’s later pornography use frequency (Time 2). Overall, the model explained 48.0% of the variance in men’s and 48.1% in women’s pornography use frequency, and 40.7% of the variance in men’s and 95.7% in women’s physical IPV perpetration.

#### Psychological IPV Perpetration

The corrected chi-square difference test (χ^2^ = 28.63, p = .053) indicated no significant difference between the unconstrained and the constrained models, suggesting that the associations do not differ significantly between men and women. Therefore, for the sake of parsimony, we reported the results of the constrained model. The fit indices of this constrained model were acceptable for the psychological IPV perpetration model, χ2(18) = 28.63, p = .053; CFI = 0.953; TLI = 0.891; RMSEA = 0.075, 90% CI [< 0.001, 0.124]. A person’s prior pornography use frequency (Time 1) was positively and strongly associated with their own later pornography use frequency (Time 2). A person’s prior psychological IPV perpetration (Time 1) was positively and strongly related to their own psychological IPV perpetration 2 years later (Time 2). No significant partner effects were observed. However, a person’s prior pornography use frequency (Time 1) was unrelated to their own and their partner’s psychological IPV perpetration 2 years later (Time 2). A person’s prior psychological IPV perpetration (Time 1) was unrelated to their own and their partner’s later pornography use frequency (Time 2). Overall, the model explained 46.3% of the variance in men’s and 47.7% in women’s pornography use frequency, and 43.9% of the variance in men’s and 72.3% in women’s psychological IPV perpetration.

#### Sexual Coercion Perpetration

The corrected chi-square difference test (χ2 = 19.69, p = .351) indicated no significant difference between the unconstrained and the constrained models, suggesting that the associations do not differ significantly between men and women. Therefore, for the sake of parsimony, we reported the results of the constrained model. The fit indices of this constrained model were excellent for the sexual coercion perpetration model, χ2(18) = 19.69, p = .351; CFI = 0.989; TLI = 0.974; RMSEA = 0.030, 90% CI [<0.001, 0.094]. A person’s prior pornography use frequency (Time 1) was positively and strongly associated with their own later pornography use frequency (Time 2). A person’s prior sexual coercion perpetration (Time 1) was positively related to their own sexual coercion perpetration 2 years later (Time 2) with a moderate effect size. A person’s prior pornography use frequency (Time 1) was positively associated with their own later sexual coercion perpetration (Time 2) with a weak effect size. Moreover, a person’s prior sexual coercion perpetration (Time 1) was negatively and weakly related to their partner’s pornography use frequency 2 years later (Time 2). Overall, the model explained 46.7% of the variance in men’s and 49.9% in women’s pornography use frequency, and 12.7% of the variance in men’s and 39.2% in women’s sexual coercion perpetration.

## Discussion

The present study used a longitudinal and dyadic design to examine the associations between pornography use frequency and IPV perpetration (i.e., physical and psychological IPV, and sexual coercion), while controlling for masturbation frequency, religiosity, and relationship length in young adult couples. Findings in mixed-sex couples indicated that men and women’s pornography use frequency was related to a higher probability of perpetrating sexual coercion 2 years later and that men and women’s sexual coercion perpetration was related to their partner’s lower pornography use frequency 2 years later. Thus, pornography use may be an important risk factor for the development of sexual coercion perpetration over time in young adult mixed-sex couples. However, men and women’s pornography use frequency was not significantly related to physical and psychological IPV perpetration and vice-versa. The sexual nature of pornography may explain why it was only related to sexual coercion perpetration. These results suggest that the link between pornography use and sexual coercion may be bidirectional and portrayed mixed-sex couple dynamics around pornography use.

### Pornography Use Frequency and Sexual Coercion Perpetration in Mixed-Sex Couples

Partly in line with our first hypothesis, a person’s pornography use frequency was only related to a higher risk of their own sexual coercion perpetration 2 years later, with no significant differences between men and women. Our result is in line with previous cross-sectional studies reporting that soldiers who used pornography, compared to those who had never used pornography, had a higher risk of perpetrating sexual IPV (Beymer et al., 2021), violent men’s problematic pornography use was associated with higher sexual IPV perpetration (Brem et al., 2021), and male adolescents’ violent pornography use was associated with a greater odd of perpetrating sexual IPV (Rostad et al., 2019). Our finding expands these cross-sectional studies showing that the association remains over 2 years and was significant for both women and men. The result supports the Sexual Script Theory (Simon & Gagnon, 1986), which suggests that sexual scripts are learned from the observation of others and exposure to mass media including pornography, and then, users may reproduce these scripts in their sexual life with their partner (Bridges et al., 2016). Considering the sexual violence of the scripts depicted even in mainstream pornography (Bridges et al., 2010; Carrotte et al., 2020), young women and men may reproduce the observed violent sexual behaviors in their sexuality with a partner as they may use pornographic scripts as a source of sexual education (Bridges et al., 2016; Sun et al., 2016). Indeed, pornography is often young adults’ main exposition to others’ sexual behaviors, and they may perceive pornography as realistic depictions of real-life sexuality (Štulhofer et al., 2012). Moreover, past work has shown that pornography use is more strongly related to verbal sexual coercion, compared to physical sexual coercion (Marshall & Miller, 2023), and only verbal sexual coercion was reported by participants at Time 2. Thus, our findings should not be extended to suggest that pornography use is related to coercive physical behaviors but rather exclusively to verbal coercive behaviors.

In an exploratory way, our findings also indicated that a person’s sexual coercion perpetration was related to their partner’s lower frequency of pornography use 2 years later, with no significant differences between men and women. Victims of sexual coercion may report lower sexual pleasure and even flashbacks during sexual activity, including solitary one, and thus avoid sexuality including pornography use (Coker, 2007). Moreover, sexual coercion includes loss of power or control in the sexual realm and the perpetrator may also exert control over the victim’s solitary sexual behaviors, leading to less frequent pornography use (Breiding et al., 2015; Coker, 2007). Our findings expand previous studies showing that the association between pornography use frequency and sexual coercion might be bidirectional.

Contrary to our second hypothesis, the associations between pornography use frequency and sexual coercion perpetration did not differ significantly between men and women. Thus, our result suggests that women’s pornography use frequency is also related to higher odds of perpetrating sexual coercion 2 years later. Most previous studies found significant pornography–IPV associations only for men (Jongsma & Timmons Fritz, 2021; Rostad et al., 2019). There might be a less marked difference between women and men in young adults as their pornography use is more frequent at this stage for both men and women (Bőthe et al., 2020; Peter & Valkenburg, 2016). Thus, even if violent behaviors depicted in pornography are highly gendered (e.g., a man dominating a woman), young adult women using pornography may also tend to reproduce these behaviors toward their partner. Some past studies reporting gendered differences examined only violent pornography, which may be more frequently used by young men that are also already perpetrating IPV (Rostad et al., 2019). However, it is also possible that our dichotomized measure of sexual coercion did not allow us to show gendered differences in the link between pornography use frequency and the severity of sexual coercion as men usually perpetrated more severe sexual coercion.

### Pornography Use Frequency and Physical and Psychological IPV Perpetration in Mixed-Sex Couples

Contrary to our first hypothesis, a person’s pornography use frequency was not significantly related to their own and their partner physical and psychological IPV perpetration 2 years later with no significant differences between men and women. In line with the Sexual Script Theory (Simon & Gagnon, 1986), even if mainstream pornography often depicted physical (e.g., spanking and slapping) and psychological (e.g., name-calling) violent content (Bridges et al., 2010), users would not reproduce these scripts outside of sexuality in their day-to-day interactions with their partner (Bridges et al., 2016). This might be due to the sexual nature of the scripts portrayed that teach one how to behave in sexual encounters only (Simon & Gagnon, 1986). Results are in line with some studies reporting that pornography use was not significantly related to physical IPV perpetration (Hatch et al., 2020; Huntington et al., 2021) and psychological IPV perpetration in men (Rostad et al., 2019). However, our results are in contrast with other studies showing that pornography use is associated with physical (Beymer et al., 2021; Brem et al., 2021) and psychological IPV perpetration (Beymer et al., 2021; Huntington et al., 2021). Discrepancies between findings might be explained by differences between samples. Indeed, these previous studies included older participants or samples that may not represent the experience of the general population (e.g., army soldiers, violence men, participants aged between 18 and 34 years old; Beymer et al., 2021; Brem et al., 2021; Huntington et al., 2021) rather than young adult couples from the community.

### Limitations and Futures Directions

The present study has some limitations that should be considered. First, although our longitudinal design examined the directionality of the associations, the lack of statistical control for all other potential confounding factors makes it impossible to determine causal relations (e.g., impulsivity, emotion dysregulation, and sexual desire). Second, our study relies only on self-reported measures, which are subjected to recall bias and social desirability. Indeed, we only used a self-reported IPV perpetration measure, which may be subject to underreporting and denial. Moreover, due to the low prevalence of sexual coercion in our sample and the low variability in some items (only verbal sexual coercion was endorsed at Time 2), sexual coercion was recoded into a binary variable, which may have reduced power and constrained our assessment of sexual coercion to verbal sexual coercive behaviors. Third, the findings’ generalizability is limited by our nonrepresentative convenience sample of young adults where self-selection biases may occur. We only recruited seven same-sex couples limiting the statistical analyses. Future studies should oversample sexual and gender diverse participants to further our preliminary results. Finally, future studies should consider the context in which pornography is used (e.g., pornography use motivations, type of pornography used) as well of specificities of young adults’ romantic relationships (e.g., conflicts resolution) to have a better understanding of the mechanisms at play in the associations between pornography use and sexual coercion perpetration.

## Conclusion

This study moved beyond previous investigations concerning the associations between pornography use and IPV by using a sample of young adult couples from the community, examining the directionality of associations using a 2-year longitudinal design, and using a dyadic design considering both partners’ pornography use and IPV perpetration. The findings suggest potential bidirectional links between pornography use frequency and sexual coercion perpetration. This highlights the relevance of addressing pornography use frequency as an important risk factor of sexual coercion perpetration in prevention and intervention efforts targeting young adult mixed-sex couples, especially during this period in life where they are learning healthy romantic relationships. This supports the Sexual Script Theory (Simon & Gagnon, 1986) that young adults are at risk to reproduce violent pornographic scripts with their partner in real life leading to sexual IPV perpetration. Pornography literacy and teen dating sexual violence prevention programs may be beneficial to young adults to help them understand that sexual behaviors depicted in pornography may represent sexual violence and are not necessarily enjoyable for their partner, as opposed to what is shown in pornography (Dawson et al., 2020).

## Supplemental Material

sj-docx-1-jiv-10.1177_08862605241234656 – Supplemental material for Associations Between Pornography Use Frequency and Intimate Partner Violence Perpetration Among Young Adult Couples: A 2-Year Longitudinal Study Supplemental material, sj-docx-1-jiv-10.1177_08862605241234656 for Associations Between Pornography Use Frequency and Intimate Partner Violence Perpetration Among Young Adult Couples: A 2-Year Longitudinal Study by Mandy Vasquez, Marie-Ève Daspe, Bőthe Beáta, Audrey Brassard, Yvan Lussier and Marie-Pier Vaillancourt-Morel in Journal of Interpersonal Violence
