# Supplementary material for: Associations Between Pornography Use Frequency and Intimate Partner Violence Perpetration Among Young Adult Couples: A 2-Year Longitudinal Study
Source: J Interpers Violence. 2024 Mar 7;39(21-22):4260–84. doi: 10.1177/08862605241234656 (PMC11465595; doi:10.1177/08862605241234656)
Supplement: sj-docx-1-jiv-10.1177_08862605241234656 – Supplemental material for Associations Between Pornography Use Frequency and Intimate Partner Violence Perpetration Among Young Adult Couples: A 2-Year Longitudinal Study [file sj-docx-1-jiv-10.1177_08862605241234656.docx]

**Supplemental Material**

**Table S1**

*Descriptive Statistics and Correlations between Pornography Use Frequency, IPV Perpetration, and Control Variables in Same-Sex Couples (n = 7)*

|  | Skew. (S.E.) | Kurt. (S.E.) | *M (SD)* | 1. | 2. | 3. | 4. | 5. | 6. | 7. | 8. | 9. | 10. | 11. |
| --- | --- | --- | --- | --- | --- | --- | --- | --- | --- | --- | --- | --- | --- | --- |
| 1. Pornography use frequency T1^a^ | 0.52 (0.60) | -0.56 (1.15) | 2.93 (2.09) | **.15** | -.23 | .25 | .27 | .18 | .03 | -.25 | .27 | -.16 | .52 | .05 |
| 2. Physical IPV perpetration T1 | 1.57 (0.60) | 0.50 (1.15) | 0.21 (0.43) | -.35 | **.65** | .20 | .47 | .76* | .45 | .14 | .65 | .25 | <.01 | -.25 |
| 3. Psychological IPV perpetration T1 | 0.84 (0.60) | -0.28 (1.15) | 1.86 (1.99) | -.73 | .73 | **.35** | .14 | .44 | .10 | .39 | .28 | -.05 | -.23 | -.46 |
| 4. Sexual coercion perpetration T1^b^ | 1.57 (0.60) | 0.50 (1.15) | 0.21 (0.43) | - | - | - | - | - | - | - | - | - | - | - |
| 5. Masturbation frequency T1 | 0.29 (0.60) | -1.11 (1.15) | 4.36 (2.13) | .84* | -.24 | -.62 | - | **.11** | .15 | -.64 | .50 | -.61 | <.01 | -.41 |
| 6. Religiosity T1 | 1.43 (0.60) | 1.94 (1.15) | 4.14 (1.51) | -.04 | .11 | .06 | - | .12 | **.86*** | -.25 | .42 | -.28 | -.25 | -.56 |
| 7. Relationship length T1 (months) | 1.28 (0.60) | 1.07 (1.15) | 38.00 (29.92) | -.25 | .14 | .39 | - | -.64 | -.25 | - | -.48 | .68 | .79 | .38 |
| 8. Pornography use frequency T2^a^ | -0.19 (0.64) | -1.06 (1.23) | 3.17 (1.90) | .90* | -.58 | -.82* | - | .76 | -.45 | .03 | **-.28** | -.08 | .37 | .22 |
| 9. Physical IPV perpetration T2^b^ | 2.42 (0.66) | 5.51 (1.28) | 0.27 (0.65) | - | - | - | - | - | - | - | - | - | - | - |
| 10. Psychological IPV perpetration T2 | 0.09 (0.66) | -2.04 (1.28) | 1.73 (1.74) | -.33 | .45 | .79 | - | -.27 | <.01 | .19 | -.50 | - | **<.01** | -.61 |
| 11. Sexual coercion perpetration T2^b^ | 3.32 (0.66) | 11.00 (1.28) | 0.09 (0.30) | - | - | - | - | - | - | - | - | - | - | - |

*Notes. M* = Mean. *SD* = Standard Deviation. Skew. = Skewness. Kurt. = Kurtosis. S.E. = Standard Error. T1 = Time 1. T2 = Time 2. IPV = Intimate Partner Violence. Correlations presented below the diagonal represent the actor associations (i.e., association between an individual X and their own Y), correlations presented above the diagonal represent the partner associations (i.e., association between an individual X and their partner Y), and correlations on the diagonal (in bold) represent between partners correlations.

^a^ 0 = never, 1 = less than 1 time per month, 2 = 1 time per month, 3 = 2–3 times per month, 4 = 1 time per week, 5 = many times per week, 6 = 1 time per day, and 7 = many times per day.

^b^ All participants reported the same value for sexual coercion perpetration at Time 1 and Time 2, and physical IPV perpetration at Time 2. As they were a constant, correlations could not be computed between them and the other variables in the analysis. **p* < .05
